# Inishowen Child and Adolescent Mental Health Service: Developing a Learning Health Microsystem

**DOI:** 10.1002/lrh2.70077

**Published:** 2026-04-05

**Authors:** Tom Foley, Bennie Steyn, Eoin Fitzgerald, Liesl Roos, Areej Gazzaz, Mohamed Mohamed, Jumanah Al Ibrahim, Amr Abu Shehab, Gavin McBridge, Aoife Robb, Sheila Gill, Caoimhe McDaid, Breidge McGarvey, Jessica Du Preez, Tom Sharpe, Paige Leigh McDonald, Sue Lacey Bryant, Fiona McNicholas

**Affiliations:** ^1^ CAMHS, Buncrana Primary Care Centre Buncrana Donegal Ireland; ^2^ University College Dublin Dublin Ireland; ^3^ Newcastle University Newcastle‐upon‐Tyne UK; ^4^ Children's Health Ireland Dublin Ireland; ^5^ Donegal Mental Health Service Donegal Ireland; ^6^ University of Galway Galway Ireland; ^7^ George Washington University Washington USA; ^8^ Manchester Metropolitan University Manchester UK

**Keywords:** access to care, complexity, learning communities, learning health systems, mental health

## Abstract

**Background:**

Learning health systems (LHSs) offer a model for continuous improvement in complex, real‐world clinical environments. While widely described internationally, little evidence exists regarding their implementation within Irish mental health services. Inishowen child and adolescent mental health service (CAMHS), a small rural microsystem with limited digital maturity, sought to apply a LHS framework through the creation of a learning community, manual data collection and a living standard operating policies and procedures (SOPP) manual.

**Aim:**

This study examines the development and early outcomes of this locally driven learning health microsystem, taking wait times as an exemplar. It explores the organizational complexity using the non‐adoption, abandonment, scale‐up, spread and sustainability (NASSS) framework.

**Methods:**

A descriptive case‐study design was used. Data were drawn from routine audits, caseload analysis, patient experience measures, referral metrics, and outputs from the learning community. The NASSS framework was applied to analyze complexity across conditions, technology, adopters, organization, wider system influences, and the capacity for embedding and adaptation over time.

**Results:**

Implementation of LHS processes was feasible despite paper‐based records and minimal infrastructure. Between 2020 and 2024, the caseload reduced by half; from 2023, 100% of young people were seen within 3 months, and maximum waiting times fell from 743 days in 2020 to 49 days in 2025. Patient and family experience remained highly positive. Several elements, including the SOPP and youth engagement, became embedded, whereas continuous data collection remained vulnerable to staff turnover and workload pressures.

**Conclusion:**

A learning health microsystem can emerge in digitally immature, resource‐constrained CAMHS settings. Inishowen CAMHS demonstrates measurable service improvements and offers a replicable early model for LHS development in Irish mental health care. Future work should focus on scaling this microsystem approach and spreading nationally, strengthening digital infrastructure to support faster, more reliable learning cycles and to sustain improvements over time.

## 1 Introduction

Across healthcare systems internationally, the persistent gap between evidence and practice continues to undermine quality and safety. Braithwaite et al. [1] found that across countries and specialties, only 60% of care, on average, aligns with evidence or consensus‐based guidelines; another 30% of care is waste or of low value and 10% of patients experience adverse events [1]. This problem, long recognized in the literature, is rooted in the recognized complex adaptive nature of healthcare systems, where outcomes arise from dynamic interactions among patients, staff, technologies, and organizational structures ([2]).

Within this context, the United States Institute of Medicine introduced the concept of the learning health system (LHS), proposing a model in which data generated through routine care are transformed into knowledge and then reapplied in practice through rapid cycles of improvement [3]. The LHS was later defined as a system in which:

“science, informatics, incentives, and culture are aligned for continuous improvement and innovation, with best practices seamlessly embedded in the delivery process, [with] patients and families (as) active participants in all elements, and new knowledge captured as an integral by‐product of the delivery experience” [4].

The lead author developed a framework for designing and evaluating LHS [5, 6]. This was followed by a toolkit for implementing the framework [7], developed in collaboration with researchers in the US, Europe and Australia [8].

A LHS is centered around a learning community [6]. The patient is central to the learning community along with clinicians who meet in a psychologically safe environment to reflect on visible data pertinent to them, discuss uncertainties, derive collective insights, and formulate responses [9]. Such communities help clinicians understand not only quantitative findings but also the nuanced realities of clinical scenarios, the complexity behind variation, and the interplay of multiple contributing factors.

Examples of LHSs have been described and implemented internationally across the full range of specialties [10]. Within Ireland, the published experience is modest but growing, with examples from general practice [11] and more recently the Irish epilepsy electronic patient records [12]. The Health Service Executive (HSE), the publicly funded health system in Ireland, Digital Health Strategic Implementation Roadmap commits to creating a Learning Health and Care System in Ireland [13].

Perhaps sparked by the very negative recent appraisal of Child and Adolescent Mental Health Services (CAMHS) in Ireland and the overall lack of electronic health record systems [14], the National Child and Youth Mental Health Office has committed to a LHS approach for CAMHS in its three‐year action plan [15].

### 1.1 Case Example CAMHS in Ireland

It is within this evolving national landscape that Inishowen CAMHS embarked on an effort to apply a LHS framework. The team is a small, semi‐rural clinical microsystem, caring for young people with moderate to severe mental health problems, with an under‐18 catchment population estimated at 12030. As of November 2025, the core Multidisciplinary Team (MDT) consisted of 1 Whole Time Equivalent Consultant Psychiatrist, 1 Non‐Consultant Hospital Doctor, 2.5 Nurses, 1 Occupational Therapist, 1 Social Worker, 0.75 Psychologist, and 2 Administrative Staff. 50% of patients have a diagnosis of Attention Deficit Hyperactivity Disorder, 37% have an Emotional Disorder, 4% have an Eating Disorder, 1% have Psychosis or Bipolar Affective Disorder, and 24% of the young people in the service are Autistic. The LHS has focused attention on a range of problems of interest over time since its launch in 2020. However, given the unnecessary suffering and multiple negative impacts that derived from lengthy waits to access CAMHS services in Inishowen, as in CAMHS elsewhere, wait times were an initial focus and are reported upon here as an exemplar of the impact of the LHS.

Importantly, this LHS development has occurred in a setting with minimal digital infrastructure, limited administrative support, and reliance on paper‐based clinical records. As such, the experience provides an opportunity to consider how learning can emerge not only from sophisticated technologies but also from human relationships, reflective practice, and simple organizational structures that allow insights to be captured and acted upon.

The development of a LHS in Inishowen CAMHS has been shaped by several challenges. The conditions managed by CAMHS are inherently complex, influenced by biological, psychological, social, and environmental determinants that lie largely outside the team's control. The service functions with limited technological support; although a digital Patient Administration System is available, all clinical documentation remains on paper, rendering data collection slow, infrequent, and labor‐intensive.

The adopters of the system, including young people, families, and clinicians, each bring their own constraints. Young people and families may face variable technological literacy, fluctuating motivation, or socioeconomic pressures that affect engagement. Staff must gather data manually, participate in the learning community, reflect on performance, and modify practice, all within heavy clinical workloads.

Organizationally, Inishowen CAMHS exists within a broader county‐wide system that has limited strategic alignment with LHS principles, slow governance processes, digital immaturity, and pressing recruitment and resource challenges. The wider health and social care landscape also presents difficulties, including funding pressures, fragmented services, and a lack of integrated pathways.

This constellation of factors created a demanding environment but also a burning platform for the development of a sustainable learning system. It was established as a practical and urgent attempt to improve the service, rather than as a research project. Formal research ethics approval was therefore not required.

## 2 Aim

Against this backdrop, Inishowen CAMHS set out to determine whether a LHS could emerge in a digitally immature environment and whether a clinical microsystem could meaningfully function as a LHS in the absence of an enabling macrosystem.

## 3 Methods

The team established a Learning Community, intended to be a safe space for reflection on team performance, with a positive error culture. All members of the MDT were members of the Learning Community. The full MDT co‐designed the Terms of Reference and participated in the Learning Community.

The Learning Community met at least every 2 months and received data collected about Inishowen CAMHS, including:

- Activity data (nationally benchmarked across Key Performance Indicators [KPIs])
- Patient and Family Reported Experience Data (using the Evaluation of Service Questionnaire)
- Verbal complaints and compliments
- Written complaints and compliments
- Feedback from Young Person and Family Advisory Groups
- Audit data (including compliance with policies, procedures and national standards)

BOX 1 **Process and Measurement.**

The Inishowen Team brought together existing policies and procedures to form a Standard Operating Procedures and Policies (SOPP) Manual, in line with the National CAMHS Operational Guideline [16]. The SOPP also contains a Learning Strategy outlining the continuous improvement cycle described in this paper. It details Learning Measures for each of the policies, including a comprehensive schedule of audits, PREMs, PROMs, complaints and compliment returns, as well as young person, family and GP advisory group feedback. These Learning Measures are collected in the Inishowen CAMHS Learning Data Workbook (a large spreadsheet). The SOPP is intended to be updated regularly and to act as a repository of the improvements that we make as a team. As such, it reflects our culture and is an important mechanism for ensuring that we do what we say we will do.

The group collectively made sense of this data, which was usually collected by the Non‐Consultant Hospital Doctor and presented by the Clinical Lead. The team also developed a Standard Operating Policies and Procedures (SOPP) manual (Box 1).

**FIGURE 1:**
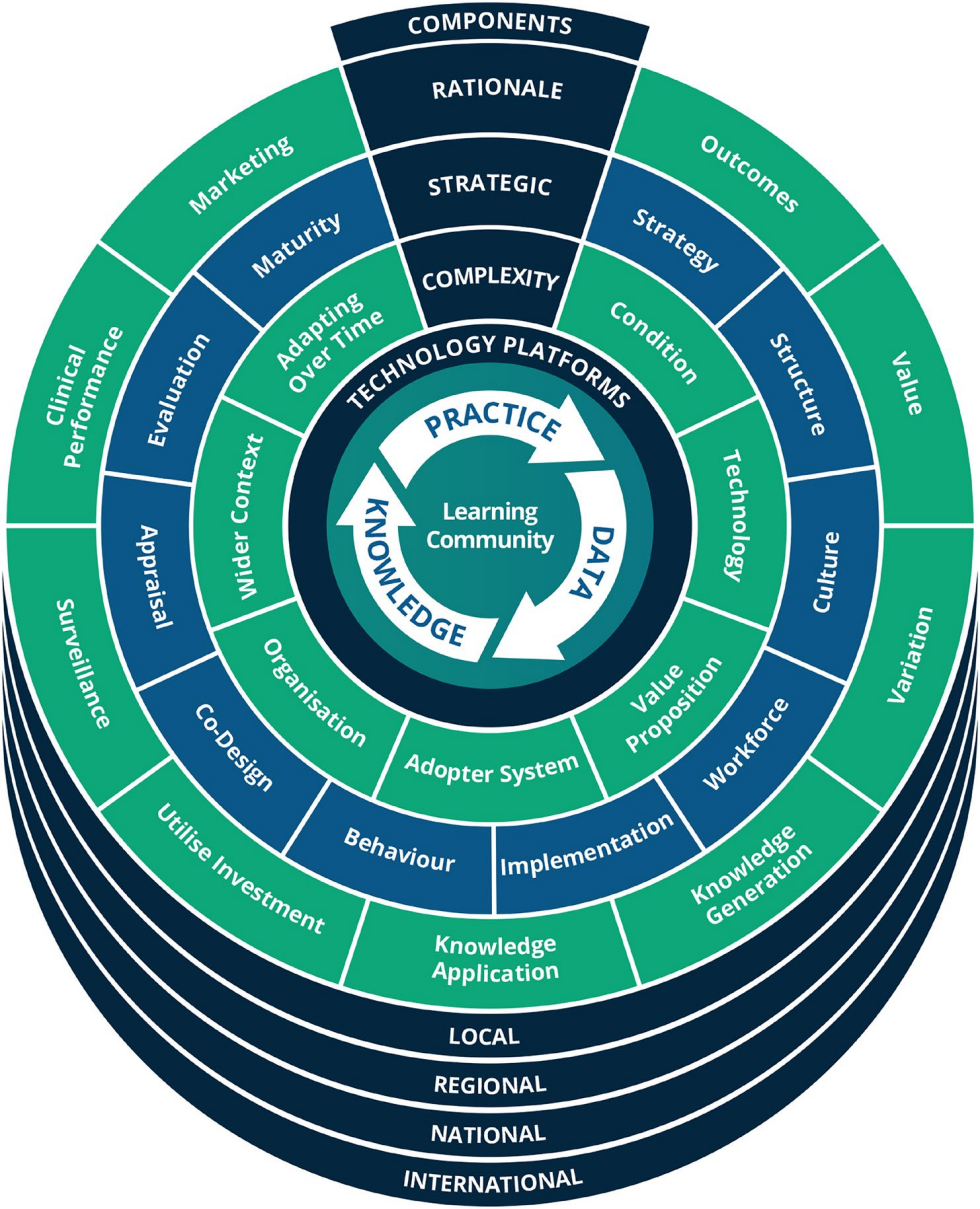
The learning healthcare project framework [6].

The Learning Healthcare Project Framework (Figure 1) [6] was employed as the underpinning conceptual model to guide the development of Inishowen CAMHS into a LHS.

The Learning Community undertook iterative cycles of reflection and improvement. This continuous series of learning cycles captured data about practice within the system, turned that data into actionable knowledge and put the knowledge back into practice to bring about an improvement in a problem of interest, such as long waiting times (Box 2) and gaps in care planning.

Through this work, the team has generated insights into what components of an LHS can be developed locally with minimal technology and where external support and infrastructure become essential.

Data is manually extracted from paper records and aggregated into a customized spreadsheet, including clinical and operational audits and Patient Reported Experience Measures (PREMS), which are conducted according to the schedule outlined in the SOPP. Basic descriptive analysis and visualization is presented in a spreadsheet. Interpretive analysis is conducted by the Learning Community, informed by local experience, national reports and international evidence.

The views of young people and families are incorporated into the LHS through a Young Person Advisory Group and a Parent Advisory Group. Verbal compliments and complaints are logged and reported to the Learning Community. The Evaluation of Service Questionnaire was used intermittently to monitor young person and parent satisfaction from September 2023.

Complexity within the system is analyzed by the lead author using the Non‐adoption, Abandonment, Scale‐up, Spread and Sustainability (NASSS) Framework [17].

In‐line with the Learning Healthcare Project Framework (Figure 1), the supporting pillars of strategy, structure, governance, culture, workforce, implementation support, behavior change and co‐design are all considered by the learning community.

## 4 Results

Between 2020 and 2025, Inishowen CAMHS established the key components of a LHS, as outlined in Figure 1. It has also demonstrated progress against local and national indicators.

### 4.1 Practice to Data

#### 4.1.1 How Do We Know What's Happening on the Ground?

In 2020, the only data being reliably captured in Inishowen CAMHS was about how long young people were waiting for care. Data about caseloads and activity levels within teams were being captured but were unreliable. There were no measures of quality or patient experience.

Since then, a systematic and comprehensive audit schedule has been established, along with caseload characteristics, process measures, Patient Reported Experience Measures, verbal complaints and compliments, and groups for engaging with young people, families, and general practitioners (GPs). All of this data is collated in the Inishowen Learning Data Workbook (a large spreadsheet) (see Box 1). Data collection remains resource intensive and relatively infrequent (6–12 monthly, depending on the item) because it must be manually extracted from paper files.

### 4.2 Data to Knowledge: How Do We Make Sense of What's Going on and Know What to Do About It?

To be useful, insights must be gleaned from collected data. Many large academic research centers have employed randomized control trials or advanced data analytics to generate quasi‐experimental results [5]. Inishowen CAMHS is a small team with no regular access to specialist data analytics. Instead, the Learning Community was used [9] to make sense of the data that had been collected and to reason about how performance could be improved. The Learning Community also took advantage of the knowledge that has been generated by recent national investigations into the quality of CAMHS provision in Ireland [14, 18, 19, 20] and international research publications and best practice guidelines [21]. Peer reviewed literature and clinical decision support tools, such as BMJ Best Practice, are provided by the HSE library through national licenses [22]. Box 2 illustrates how this approach was applied to reducing the time that young people spent waiting for services.

BOX 2 **A Case Study of Impact: Improving Access to CAMHS.**

From 2020, the Learning Health System approach was applied in an attempt to reduce the waiting list. The Learning Community reviewed the limited available data and used their experience of the system to understand why the waiting list was so long. Their insight was that the waiting list was only a symptom of underlying problems with flow in the system, such as a lack of intensive and specialist interventions and inappropriate referrals. The resulting long waiting list created a vicious cycle by delaying treatment and making recovery more difficult.

A suite of initiatives was implemented, primarily to reduce the caseload and increase the intensity of interventions. Projects were undertaken to establish a General Practitioner liaison group, to improve working with related services, to provide access to digital interventions and self‐help materials, and to develop new ADHD and Eating Disorder pathways. Perhaps most impactful was to form a network of community and voluntary therapy providers to work more intensively with CAMHS patients.

Repeated learning cycles identified some failed initiatives, but the overall result was that in 2024, the caseload was half what it had been in 2020 and Inishowen was the only CAMHS team in Ireland to have seen 100% of patients within 3 months.

Despite year‐on‐year increases in referrals and chronic staffing shortages, most waits are now measured in days rather than months or years. In October 2020 young people in Inishowen had been waiting up to 743 days for a first appointment. By November 2025, the longest wait was 49 days. This is likely to have avoided unnecessary suffering and been associated with significant economic benefits including the avoidance of missed education, crisis presentations and the requirement for more intensive interventions. This early access to therapeutic interventions allowed for faster patient recovery, and earlier discharge from the service, which further reduced the caseload.

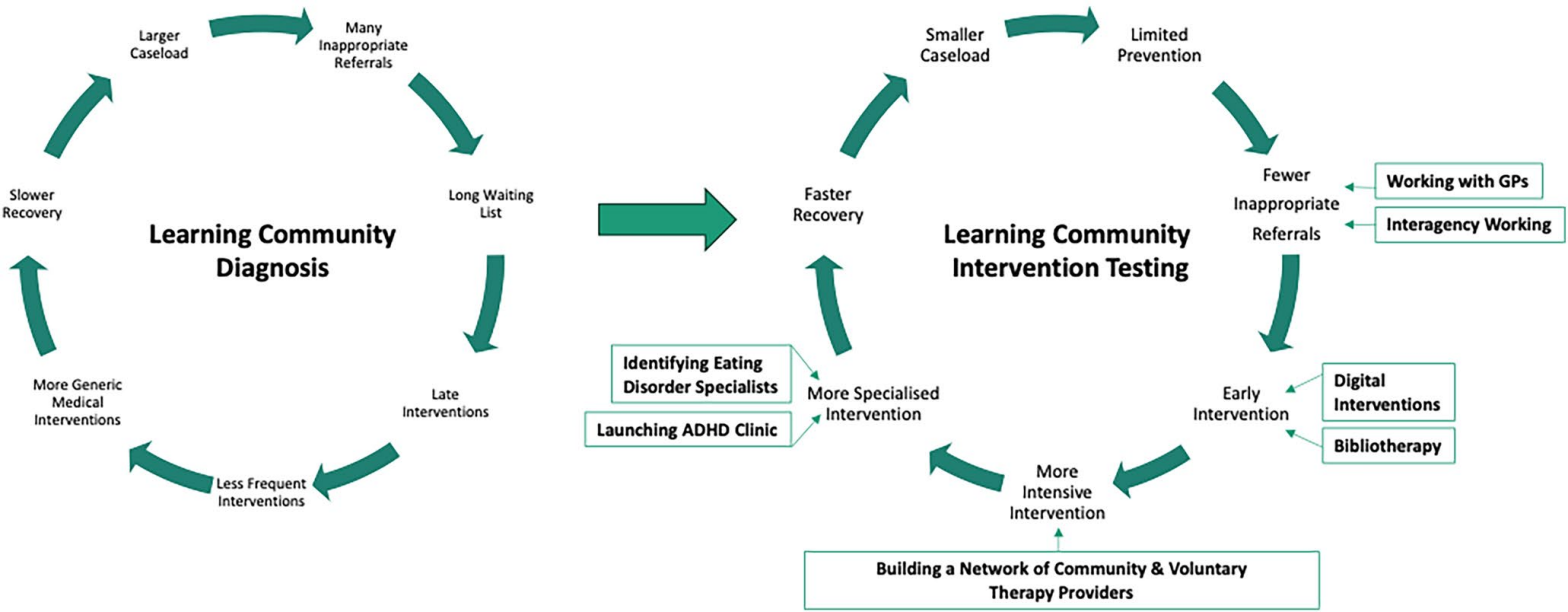

### 4.3 Knowledge to Practice

#### 4.3.1 How Do We Make the Change?

Knowledge generated through research is often published in academic papers, books, and guidelines. It is passed on through formal education and training or through informal networks and even word of mouth among clinicians. These are important but not always fast or reliable methods of knowledge mobilization. Knowledge generated through local experience, such as serious incident reviews and root cause analysis, often lack systems to ensure that learning is shared and recommendations acted upon effectively [23]. The improvement cycles of a LHS rely on a means of rapidly putting knowledge generated from local experience or international evidence into practice.

Large LHSs often involve a knowledge repository, carefully curated by a knowledge specialist or librarian [24], with a team of behavior change experts to devise effective interventions [25]. Again, Inishowen CAMHS does not have access to such expertise. Instead, the team has developed a Standard Operating Policies and Procedures (SOPP) manual to act as its knowledge repository (see Box 1). This is intended to be a living document. When the Learning Community generates new actionable knowledge, for example a new approach to Individual Care Planning, the SOPP is updated to describe the new process and how its successful implementation should be measured. This is in contrast to the historical practice of reviewing policies after a predefined number of years. It is hoped that the Learning Community's ownership of the SOPP encourages compliance with its processes, but where this fails, the issue is identified through the learning measures and audits.

### 4.4 Understanding the Complexity Within Inishowen CAMHS

The Learning Healthcare Project Framework, outlined in the methodology section (Figure 1), highlights that the improvement cycle outlined above—Practice to Data to Knowledge to Practice—is necessary but not sufficient to deliver a successful LHS. Many innovations have failed to deliver the intended results because of uncontrolled complexity in the system [17].

A complex system such as a CAMHS is characterized by a large number of interacting elements, in which minor changes can produce disproportionate and unintended consequences [26]. Elements evolve with one another and with the environment in an irreversible way. That may appear predictable in retrospect, but is in fact unpredictable prospectively.

In Inishowen CAMHS, an adapted version of the Non‐adoption, Abandonment, Scale‐up, Spread and Sustainability Framework [5, 17] was used to analyze complexity within the system. The following domains of complexity are notable.

#### 4.4.1 The Conditions

The conditions/disorders managed by CAMHS are generally complex, involving biological, psychological, social, and economic determinants, many of which are beyond the control of the CAMHS team.

#### 4.4.2 Technology

The lack of modern information systems within Inishowen CAMHS makes the collection and use of data difficult and slow.

#### 4.4.3 Adopters

**CHART 1:**
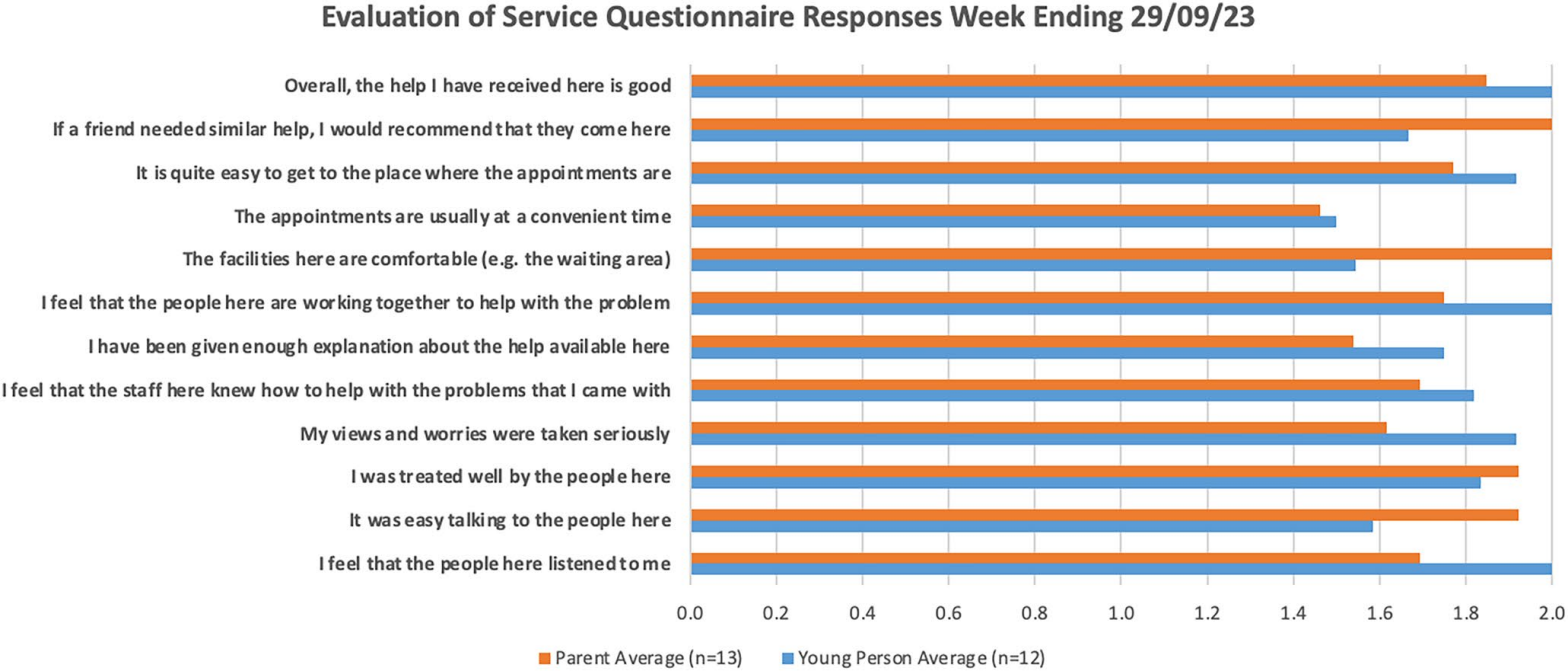
First evaluation of service questionnaire snapshot (0 = “*Not true*,” 1 = “*Partly true*,” and 2 = “*Certainly true*”).

Those who need to adopt our systems are impacted by complexity:

- *Young People and Families*: May have limited digital and health literacy, variable insight and motivation, and may be under extreme socioeconomic stress, making it difficult to engage with services (see Box 3). Despite these challenges, patient satisfaction was measured from 2023 and was very positive (Chart 1).
- *Staff*: Face challenges to gather data, participate in the Learning Community, reflect on team performance and change practice. In some cases, these factors can represent a threat to professional identity and can challenge underlying cultural assumptions. No staff satisfaction surveys were conducted because of small numbers, but it is a crude measure of engagement that most of the team co‐authored this paper.

#### 4.4.4 The Organization

Inishowen CAMHS is a small part of a larger county‐wide mental health service that has funding, recruitment, and regulatory pressures and a limited recent history of proactive quality improvement initiatives or applied research participation. There is no regional strategic alignment with the proposed LHS approach, which represents a significant change to the current centralized organizational processes and culture. CAMHS remains poorly integrated with key partner services, such as Primary Care Psychology and Child Disability Teams, which are managed under different parts of the organization.

#### 4.4.5 The Wider System

There have been challenges in the wider health system, including funding pressures and recruitment freezes. However, there is also significant organizational, public and political pressure for improvement within CAMHS. There is strategic alignment with the National Office for Child and Youth Mental Health Services [15], The Digital Health Strategic Implementation Roadmap [13], and the Health Research Board National Mental Health Research Strategy [27]. The approach is also in line with international evidence on system‐wide improvement approaches [28].

#### 4.4.6 Embedding and Adaptation Over Time

Some elements of the LHS approach, such as the SOPP and patient engagement, have become embedded within Inishowen CAMHS. Other aspects, such as continual data collection, require significant effort and it is unclear how they would be sustained if there was significant turnover in the team.

BOX 3 **Failure.**

Many of the initiatives considered by the Learning Community have not worked, often because of the issues highlighted in the complexity section of this paper. Sometimes these initiatives do not get started; other times, money and effort are spent on things that don't work. For example, two evidence‐based online groups for anxiety and depression were run by a partner agency. The Learning Community thought they would take people off the waiting list and promote recovery. Only half of the referred patients turned up to the first session and most of the rest dropped out. On another occasion, an online digital intervention for children and parents with anxiety was offered. Again, most of the patients dropped out. On yet another occasion, the team tried to adopt an online portal from an English Mental Health Trust, but after months of effort they could not get through the cybersecurity hurdles. At other times, clinically effective initiatives have failed because of a lack of financial or organizational capacity.

The LHS does not suggest that teams only try interventions that are guaranteed to work. In the Inishowen CAMHS LHS, the Learning Community accepts that success is often unpredictable and designs interventions to be “safe to fail” rather than “fail safe” [10]. Data collection and learning cycles are designed to keep track of what is happening in the system and to recognize when something is failing. Currently, the Learning Community has only limited autonomy to remove resources from failing initiatives and redirect them to what is working.

### 4.5 Supporting Pillars for the Inishowen CAMHS Learning Health System

In order to enable continuous improvement cycles in the context of the complexity challenges identified in the previous section, Inishowen CAMHS has identified the importance of the following supporting pillars from the Figure 1 framework:

#### 4.5.1 Strategy, Structure, and Governance

The SOPP contains a Learning Strategy (Box 1) and a Terms of Reference developed by the Learning Community. The broader county‐wide mental health service does not currently have a strategy to align with; however, the LHS is aligned with the 3‐year operational plan that was recently published by the National Child and Youth Mental Health Office [15]. Local governance systems are slow to adapt outside of a crisis. For example, it took 2 years for the local mental health services governance group to sign off the first version of the SOPP. By this time, it had been updated many times by the Learning Community.

#### 4.5.2 Culture

A LHS requires a culture of learning, reflection, innovation, patient safety, psychological safety, and continual improvement that has not always been universal in CAMHS nationally [14] or mental health services locally. Organizational culture can be viewed in three layers [29]:

- *Visible Manifestations*: The distribution of services and roles, the physical environment, pathways of care, staffing practices, reporting arrangements, policies and procedures.
- *Shared Ways of Thinking*: Values and beliefs used to justify the visible manifestations
- *Deeper Shared Assumptions*: Unconscious underpinnings of day‐to‐day practice, such as assumptions about the role of patients, carers, and other professionals.

Culture change is slow and difficult. Within Inishowen CAMHS it has largely been achieved through the visible manifestations. The SOPP has become a powerful culture change tool. It is owned, updated, and monitored by the Learning Community and it explains how things are done around here. The Young Person's Advisory Group has also had an impact on the visible manifestations, as for example, they have redesigned the waiting area and created artwork to change the look and feel of the clinic area.

The culture of Inishowen CAMHS also evolves as team members join and leave. This can be an unsettling experience for the team and for new joiners, and a new Induction Pack is under development, with the aim of helping to convey the team culture and practices to new joiners.

#### 4.5.3 Workforce

Like most CAMHS in Ireland, Inishowen has not reached recommended staffing levels. The local team has had little input into the prioritization of vacancies or their recruitment. Staff have been moved in and out of the team without consultation or regard to team stability, as the local management team has tried to balance competing demands and risks in other services. This makes service planning, targeted recruitment, and improvement more difficult and increases complexity.

The small size of the team means that most staff are managed by line managers who are based outside the team. This has the benefit of ensuring that each team member has a manager from their own profession, but it can also make it difficult to standardize policies and procedures. Historically, it has encouraged differing cultures of practice and approaches to performance management. This has occasionally led to the perception of conflicting values within the team. In this system, cross‐discipline leadership becomes distributed by default, rather than by design.

In recent years, the effective workforce has grown to include partners from community and voluntary organizations (see Box 3). This has added resilience and enabled the team to continue providing a timely service. It has also increased complexity and resulted in frequent funding and approval crises, due to a lack of budgetary autonomy and single year budgets.

#### 4.5.4 Implementation Support

A LHS may improve outcomes, experience, and value, but it is not free. It requires significant project management and administration. This burden currently falls largely on clinical staff. Progress has been limited by a lack of dedicated project and business support to release clinical time for patient care. This has reduced its resilience and made it more vulnerable to key staff leaving.

#### 4.5.5 Behavior Change

Many successful LHSs employ an evidence‐based approach to behavior change [6]. This often starts with an understanding of the drivers of the behavior [30]. These drivers are then linked to evidence‐based interventions for staff, patient and the organization. These can include, intervention functions such as, education, persuasion, incentivization, restriction, training, modeling, and enablement, among others [25]. The structure and governance of Inishowen CAMHS means that most of the intervention functions are not available within the team—for example, education, training, restriction, and incentivization are managed by line managers outside the team and are rarely coordinated. Behavior change in Inishowen CAMHS is largely achieved through persuasion and modeling. These are often sufficient because of the current positive culture but the literature suggests that they are not appropriate in all potential scenarios [25].

#### 4.5.6 Co‐Design

Co‐design is central to a LHS and is embedded in the Inishowen CAMHS approach (see Box 2). Staff are involved through the Learning Community; major changes are discussed at the patient and parent advisory groups and at the GP liaison group. There is scope to improve this further by adding young person and family representation to the Learning Community.

## 5 Discussion

Although digital immaturity, system fragmentation and resource pressures created a challenging environment in which to build and test a sustainable learning system, it simultaneously provided opportunities to strengthen core learning practices by engaging directly with real‐world complexity, interpreting data as it arose, and continually reshaping understanding and processes in response.

Furthermore, Inishowen CAMHS possesses distinct strengths that support the emergence of a microsystem‐level LHS. Its small scale enables close communication, rapid feedback, and collective oversight of new initiatives. Relationships within the team are strong and collaborative, creating a culture of trust and psychological safety that supports reflective practice. The service also benefits from positive relationships with external partners, including GPs, schools, and community organizations.

The team is also able to motivate staff through shared purpose and visible improvement, and because processes such as the SOPP are developed collectively, they carry a sense of ownership that supports adherence. These strengths indicate that a clinical microsystem, particularly one characterized by close‐knit relationships and a strong learning culture, may be well positioned to adopt LHS principles even without the technological or structural supports typically associated with large‐scale learning systems.

Despite these achievements, challenges remain. The SOPP has been a useful repository of knowledge generated locally, nationally, and internationally, but it contains primarily operational guidance. There is a need for a similar clinically focused repository. Similarly, most of the learning measures collected are process‐focused. Learning could be enhanced through the deployment of Patient Reported Outcome Measures.

The service is small and does not have autonomy over its budget, recruitment, retention, or line management. This can make it more difficult to implement changes decided upon by the Learning Community.

Further improvements to the LHS infrastructure are planned and national programs might result in further progress.

### 5.1 Locally

- The team has been awarded a grant to map each of the core clinical pathways through a joint academic‐clinician‐patient co‐design event, creating a clinical counterpart to the SOPP.
- The team has been awarded another grant to create a patient reported outcome measurement framework covering those clinical pathways.
- An automated patient reported outcomes measurement platform has been procured.
- The team has partnered with the Child Outcome Measurement Consortium to train all staff on measurement‐based care.
- The team has developed research links with national and international universities, specializing in LHSs.

The team has made the case for some devolution of powers, from the regional to the local level. These include a small flexible budget for service level innovation, dedicated project management support, authority to make certain decisions locally, and the opportunity for every member of staff to ring‐fence up to 20% of time for service improvement or applied research.

### 5.2 Nationally

The team expects to benefit from several national initiatives. A National Electronic Health Record for CAMHS is under development and members of the team have become involved in its design. This will reduce the burden of data collection and near‐real‐time reporting and dashboards will shorten learning cycles. The enablement of a LHS is an explicit goal of that EHR program.

The National Child and Youth Mental Health Office has adopted a LHS approach, and the Health Research Board has launched a National Research Strategy for Mental Health that promotes applied research.

### 5.3 An Applied Research Centre

In the longer term, these developments could culminate in the establishment of an applied research center to support the LHS. The focus would be on assessing international evidence‐based interventions, as well as local innovations, in the Irish context. This could build on existing links with University College Dublin, Galway University, Ulster University, Newcastle University, Manchester Metropolitan University, and George Washington University. The approach could be replicated in other CAMHS, turning them into a network of living labs that could assess many different interventions simultaneously.

## 6 Conclusion

The experience of Inishowen CAMHS shows that a Learning Health Microsystem can develop even with limited digital infrastructure and inconsistent organizational support. Significant improvements in waiting times and strong patient and family experience demonstrate that meaningful learning cycles can occur when relationships, shared purpose, and simple structures—such as a learning community and a living SOPP—are in place.

At the same time, the work highlights the need for better digital systems, more reliable access to real‐time data, and protected improvement time if progress is to be sustained and scaled. Despite these constraints, Inishowen illustrates how a small team can test ideas rapidly, reflect collectively, and adapt care processes, offering a model that could be replicated in the broader mental health system and in other specialties.

The foundations laid in Inishowen could contribute to a more integrated national learning system. Ultimately, the case shows that becoming a LHS is a journey and that even modest beginnings can lead to meaningful transformation when supported by curiosity, collaboration, and a commitment to continual improvement.

## Conflicts of Interest

The authors declare no conflicts of interest.

## Data Availability

The data that support the findings of this study are available from the corresponding author upon reasonable request.
